# Vitamin D Deficiency in Elderly With Diabetes Mellitus Type 2: A Review

**DOI:** 10.7759/cureus.12506

**Published:** 2021-01-05

**Authors:** Ioannis Papaioannou, Georgia Pantazidou, Zinon Kokkalis, Neoklis Georgopoulos, Eleni Jelastopulu

**Affiliations:** 1 Orthopedics, General Hospital of Patras, Patras, GRC; 2 Otolaryngology - Head and Neck Surgery, General Hospital of Patras, Patras, GRC; 3 Orthopedics, University of Patras, Patras, GRC; 4 Endocrinology, Diabetes and Metabolism, University of Patras, Patras, GRC; 5 Department of Public Health, University of Patras, Patras, GRC

**Keywords:** elderly population, diabetes mellitus type 2, vitamin-d deficiency, vitamin d supplementation, diabetic complications

## Abstract

Diabetes mellitus type 2 (T2DM) is an emerging public health issue with high prevalence among older adults (>60 years old). Taking into consideration the great increase in the elderly population (approximately 7.5 billion worldwide), we can easily understand the impact of this chronic disease and its complications. On the other hand, vitamin D deficiency (VDD) is also a serious public health problem with significant impacts and multiple health effects. The correlation between DM and VDD has been suggested and established from many observational studies, reviews, and meta-analyses.

The literature in PubMed and Google Scholar was searched for relevant articles published up to October 2020. The keywords used were the following: vitamin D deficiency, elderly, and diabetes mellitus type 2. Among the 556 articles retrieved, 90 full texts were eligible and only 34 studies (12 retrospective studies, two prospective cohorts, three meta-analyses, seven cross-sectional studies, nine randomized control trials (RCTs), and one observational study) met the inclusion criteria for the review. The author's name, year of publication, country, type of study, and the number of patients were reported.

According to this review there is adequate evidence to support the correlation between VDD and T2DM in the elderly. The results from the RCTs are more conflicting and more studies are needed to confirm the impact of vitamin D deficiency (VD) supplementation on metabolic and lipid profile, oxidative stress, and the complications of T2DM in older patients. VDD is clearly related with severe retinopathy, diabetic peripheral neuropathy, and poor cognition performance, while there is consensus about the beneficial effect of VD on peripheral artery disease, foot ulceration prevention, and wound healing. On the other hand, there is controversy about the effect of VD supplementation on cardiovascular adverse events, endothelial function, and estimated glomerular filtration rate (eGFR). Finally, the association of VDD with fragility fractures and depression in the elderly with T2DM is currently insufficiently studied and remains controversial.

## Introduction and background

Diabetes mellitus type 2 (T2DM) is an emerging public health issue with high prevalence among older adults (>60 years old). The main causes are increased life expectancy and lifestyle alterations [1]. It is estimated that 20% of elderly worldwide suffer from T2DM, while a similar proportion has undiagnosed T2DM [1]. Taking into consideration the great increase in the elderly population (approximately 7.5 billion worldwide), we can easily understand the impact of this chronic disease and its complications [1]. On the other hand, vitamin D deficiency (VDD) constitutes a significant public health problem with great impact (1 billion people in 2008 worldwide) and multiple health effects (skeletal and extra-skeletal) [2]. VDD is related to many chronic diseases, such as cognition, depression, osteoporosis, cardiovascular disease, hypertension, diabetes, and cancer [3]. It is worth noting that age over 50 years is considered among risk factors for VDD [3]. Malabsorption, reduced renal function, skin decline, reduced time outdoors, and some medications (anticonvulsants) are the main factors, which predispose the elderly to VDD and its consequences [2]. The correlation between DM and VDD has been suggested from many observational studies, although the establishment of this knowledge has been done by a systematic review and meta-analysis by Pittas et al. in 2007 [4]. The main pathophysiologic mechanisms that correlate VDD with DM are dysregulation of pancreatic beta-cell function, impaired insulin sensitivity, and increased systemic inflammation [4]. In the last two decades there has been expanding evidence describing the implications of VDD in patients with T2DM, although until now no review or meta-analysis concerning the impact of VDD in the elderly with T2DM has been conducted. The aim of this study is to fill this gap in the current literature concerning this age group.

## Review

Eligibility according to PICOS (Population, Intervention, Comparison, Outcomes and Study) criteria

Type of Study

All articles in English language assessing the correlation of vitamin D deficiency (VDD) and diabetes mellitus type 2 (T2DM) in the elderly population (>60 years old), the implications of VDD in elderly with T2DM, and the current evidence of vitamin D supplementation effects in this specific age group up to October 2020 were eligible. Studies were eligible if they were longitudinal (prospective or retrospective studies, prospective cohort, case-control, cross-sectional, randomized control and meta-analysis), and if they reported any association (complications) of VDD in elderly with T2DM such as cardiovascular disease, nephropathy, retinopathy, peripheral artery disease, cognition, neuropathy, fragility fractures, depression, and quality of life. Furthermore, observational studies which correlate VDD with elderly with T2DM and randomized control studies, which describe the effects of vitamin D supplementation in this specific patient group were also eligible in this review. We complemented the search by manual scanning of reference lists of identified articles. The search was limited to studies conducted in humans.

Exclusion Criteria

Studies with subjects with mean age below 60 years old were excluded from this review. Case series, case reports, animal studies, and studies published in languages other than English were also excluded.

Type of Participants

Elderly (>60 years old) with T2DM and VDD. The research was up to October of 2020.

Type of Outcome Measures

We included studies if they measured at least one of the following: correlation of VDD with elderly patients with T2DM, effects of vitamin D supplementation in elderly with T2DM, complications of VDD in this patient group such as nephropathy, retinopathy, peripheral artery disease, cognition, neuropathy, fragility fractures, depression, quality of life, and cardiovascular diseases.

Information Sources and Search Methods

A systematic manual search was conducted in PubMed (including ahead of print and Epub) and the first 100 articles of Google Scholar databases published up to October of 2020. The keywords used were the following: vitamin D deficiency, elderly, and diabetes mellitus type 2. Among the 556 articles retrieved, 90 full texts were eligible, and only 34 studies (12 retrospective studies, two prospective cohorts, three meta-analyses, seven cross-sectional studies, nine randomized control trials, and one observational study) met the inclusion criteria for the review. The author's name, year of publication, country, type of study, and the number of patients were reported. Titles and abstracts were screened by the author and full texts retrieved for the manuscripts found relevant for the topic and excluded clearly irrelevant articles. Additional articles were searched and identified through hand searching of the bibliography. The retrieved full-text articles were assessed for eligibility for inclusion and data were extracted by the authors. Any disagreement in the selection of articles and data was solved by consensus.

**Figure 1:**
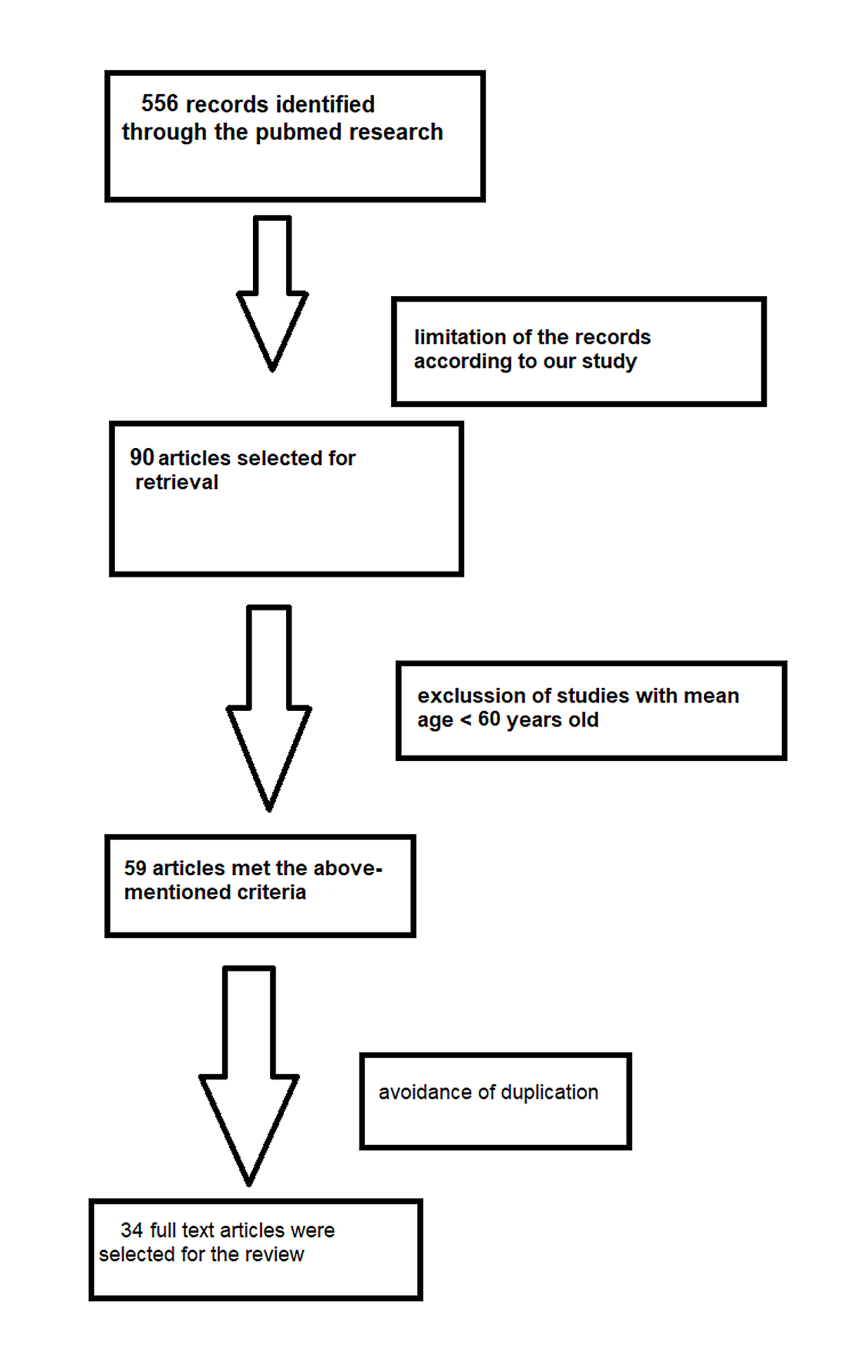
The different phases of the literature research.

The different phases of the literature search are illustrated in Figure 1.

For the assessment of study quality, the risk of bias assessment according to Newcastle-Ottawa was applied (Table 1).

**Table 1:** Quality of the selected studies by using the Newcastle-Ottawa scale.

Author	Selection	Compatibility and outcome	Total score
Wang et al [5]	4	3	7
Lucato et al [6]	4	3	7
Dalgard et al [7]	4	3	7
Calvo-Romero et al [8]	4	4	8
Kositsawat et al [9]	4	4	8
Hurskainen et al [10]	4	3	7
Zoppini et al [11]	4	4	8
Lemieux et al [12]	4	5	9
Wenclewska et al [13]	4	5	9
Fagundes et al [14]	4	3	7
Li et al [15]	4	4	8
Kampmann et al [16]	4	5	9
Witham et al [17]	4	5	9
Herrmann et al [18]	4	5	9
Samefors et al [19]	4	3	7
Chen et al [20]	4	3	7
Muscogiuri et al [21]	4	4	8
Sugden et al [22]	4	5	9
Angellotti et al [23]	4	5	9
Yuan et al [24]	4	3	7
Feldkamp et al [25]	4	4	8
Razzaghi et al [26]	4	5	9
Dall’Agnol et al [27]	4	3	7
Peng et al [28]	4	4	8
De Boer [29]	4	5	9
Suzuki et al [30]	4	3	7
Long et al [31]	4	4	8
Zhang et al [32]	4	5	9
Niu et al [33]	4	4	8
Rui-hua et al [34]	4	4	8
Westra et al [35]	4	3	7
Alcubierre et al [36]	4	4	8
Kim et al [37]	4	3	7
Perez‑Diaz et al [38]	4	3	7

Results

A total of 556 studies were identified through the database search. Out of the 90 full texts retrieved, only 34 were included in the review (12 retrospective studies, two prospective cohorts, three meta-analyses, seven cross-sectional studies, nine randomized control trials, and one observational study), 14 were published in Europe, three in the USA, nine in China, two in Brazil and one each in Japan, Korea, Canada, Mexico, Iran, and Australia. A total of 62,381 patients were included in the review.

Most of the retrospective studies (10/12) revealed a high rate of VDD in elderly patients with T2DM and a strong correlation of VDD with many complications of T2DM in this patient group, such as diabetic foot ulceration, nephropathy, retinopathy, and cognition dysfunction. Although, a retrospective study conducted in 2016 didn't reveal a direct effect of VD on coronary heart disease and the last one published in 2015 didn't show any correlation between VD and hemoglobin A1c (HbA1c) or fragility fractures.

The results from the randomized control trials (RCTs) are conflicting, although most of them support that VD supplementation improves metabolic parameters (lipids, insulin resistance, glucose) and reduces also the complications of T2DM in elderly patients. In more detail, five out of nine RCTs recorded a positive impact of VD supplementation on metabolic profiles of the elderly with T2DM and also reduction of complications associated with T2DM such as improvement of endothelial function, wound healing in diabetic foot ulceration, reduction of microvascular and macrovascular disease events, improvement of blood pressure and b-type natriuretic peptide levels. On the other hand, two relatively small RCTs suggest that VD supplementation didn't improve metabolic profile, while the remaining two RCTs revealed no impact of VD in cardiovascular risk reduction and estimated glomerular filtration rate (eGFR) improvement.

The three meta-analyses included in this review concerning different aspects of this topic were all in agreement that VDD is associated with higher risk for T2DM development, VD supplementation reduces insulin resistance, and VDD is a risk factor for diabetic peripheral neuropathy.

Six out of seven cross-sectional studies concluded that VDD is correlated with many complications of T2DM in the elderly population. These complications are the following: myocardial dysfunction, peripheral artery disease, nephropathy, neuropathy, self-reported quality of life, and vertebral fracture risk. Only one cross-sectional study didn't manage to settle VDD as a contributing factor of depression in elderly with T2DM.

Regarding the prospective studies, one of them supported that daily VD supplementation improves metabolic profiles and oxidative stress of elderly with T2DM, while the second study concludes that VDD is associated with increased risk of cardiovascular morbidity and mortality in these patients.

Finally, the unique observational study included in this review highlights that VDD is related with microvascular complications, insulin usage, and fragility fractures.

Table 2 summarizes the correlations and results between T2DM and VDD in the elderly population.

**Table 2:** The correlations and results between diabetes mellitus type 2 and vitamin d deficiency in the elderly population. VDD: vitamin D deficiency, VD: vitamin D, T2DM: type 2 diabetes mellitus, FFs: fragility fractures, HbA1c: hemoglobin A1c, RCT: randomized control trial, IR: insulin resistance, BP: blood pressure, BNP: b-type natriuretic peptide, CHD: coronary heart disease, CVS: cardiovascular, PAD: peripheral artery disease, DFU: diabetic foot ulcer, GFR: glomerular filtration rate, DN: diabetic nephropathy, DPN: diabetic peripheral neuropathy.

Author	Year	Country	Study type	Patients	Results
Wang et al [5]	2018	China	Retrospective study	785	High rate of VDD in patients with T2DM. PTH is a risk factor for FFs.
Lucato et al [6]	2017	Italy	Meta-analysis	28.258	VDD is associated with higher risk for T2DM development.
Dalgard et al [7]	2011	Faroe Islands	Retrospective study	668	VD sufficiency protects against T2DM development.
Calvo-Romero et al [8]	2015	Spain	Retrospective study	103	VDD is associated with T2DM and inverse correlations exist between VD status and metabolic control and insulin resistance.
Kositsawat et al [9]	2015	USA	Retrospective study	2.193	VDD is associated with abnormal HbA1c.
Hurskainen et al [10]	2012	Finland	Retrospective study	1.756	VDD is associated with impaired glucose and insulin metabolism.
Zoppini et al [11]	2013	Italy	Retrospective study	715	Inverse correlation of low VD and HbA1c.
Lemieux et al [12]	2019	Canada	RCT	96	VD supplementation for 6 months increases insulin sensitivity and b-cell function in individuals with newly diagnosed T2DM.
Wenclewska et al [13]	2019	Poland	RCT	92	Daily 2000 IU intake of VD for three months reduced oxidative stress and improved metabolic parameters (IR, glucose, lipids) in T2DM patients.
Fagundes et al [14]	2019	Brazil	Prospective study	75	Daily 4000 IU intake of VD for 8 weeks improved glycemic, lipid and hepatic profiles. Oxidative stress was also reduced in T2DM patients.
Li et al [15]	2018	China	Meta-analysis	2.703	VD supplementation reduces insulin resistance in T2DM patients.
Kampmann et al [16]	2014	Denmark	RCT	16	High dose of VD supplementation in T2DM patients didn’t improve the metabolic profile.
Witham et al [17]	2010	United Kingdom	RCT	61	High dose of VD supplementation in T2DM patients improved BP and BNP levels, but not the IR, the HbA1c and endothelial function.
Herrmann et al [18]	2015	Australia Finland New zeland	RCT	9.795	VDD is associated with microvascular and macro vascular disease events in T2DM patients.
Samefors et al [19]	2017	Sweden	Prospective study	761	VDD is associated with increased risk of cardiovascular morbidity and mortality in T2DM patients.
Chen et al [20]	2014	China	Cross sectional study	95	VDD is associated with myocardial dysfunction in T2DM patients.
Muscogiuri et al [21]	2016	Italy	Retrospective study	698	VD doesn’t have a direct effect on CHD but may have an indirect effect mediated by CVS risk factors such as diabetes duration, age, and sex.
Sugden et al [22]	2008	United Kingdom	RCT	87	VD supplementation with one large dose improves endothelial function in T2DM patients.
Angellotti et al [23]	2019	USA	RCT	127	VD supplementation with 4000 IU/day did not affect lipid profile and CVD risk in patients with stable T2DM.
Yuan et al [24]	2019	China	Cross sectional study	1.018	VDD is associated with increased risk of PAD in T2DM patients.
Feldkamp et al [25]	2018	Germany	Retrospective study	104	VDD is associated with increased risk of diabetic foot ulcer syndrome.
Razzaghi et al [26]	2017	Iran	RCT	60	VD supplementation for 12 weeks among patients with DFU improved wound healing, oxidative stress, metabolic and lipid profiles. .
Dall’Agnol et al [27]	2020	Brazil	Cross sectional study	114	VDD is associated with decreased GFR in patients with T2DM.
Peng et al [28]	2015	China	Retrospective study	448	Patients with T2DM with DN had lower VD levels. Status of VD can be used to identify patients at increased risk of developing nephropathy complications.
De Boer [29]	2011	USA	RCT	1.312	VD supplementation resulted in no significant difference in change in e GFR at 5 years
Suzuki et al [30]	2006	Japan	Observational study	581	VDD is associated with microvascular complication and insulin usage. VDD and insulin treatment is associated with FFs.
Long et al [31]	2017	China	Retrospective study	842	VDD is associated with severe diabetic retinopathy
Zhang et al [32]	2019	China	Meta-analysis	2814	VDD is associated with DPN. VDD is a risk factor for DPN in T2DM patients.
Niu et al [33]	2019	China	Cross sectional study	1461	VDD is associated with DPN and might be used as a predictor of DPN in elderly with T2DM.
Rui-hua et al [34]	2019	China	Retrospective study	173	VDD may predict poor cognitive performance in patients with T2DM.
Westra et al [35]	2017	Netherlands	Cross-sectional study	527	VDD appeared not to be a contributing factor to higher depression scores in people with T2DM.
Alcubierre et al [36]	2015	Spain	Cross-sectional study	292	VDD is associated with self-reported diabetes treatment satisfaction and the diabetes-specific quality of life in patients with T2DM.
Kim et al [37]	2013	Korea	Cross-sectional study	341	VD levels below 20 ng/mL were associated with an increased vertebral fracture risk in men with T2DM.
Perez‑Diaz et al [38]	2015	Mexico	Retrospective study	110	No correlation was found between VD and HbA1c or FFs.

According to this review there is adequate evidence to support the correlation of VDD with T2DM in the elderly. The results from the RCTs are more conflicting and more studies are needed to confirm the impact of VD supplementation on metabolic and lipid profiles, oxidative stress, and the complications of T2DM in older patients. VDD is clearly related with severe retinopathy, diabetic peripheral neuropathy, and poor cognition performance, while there is consensus about the beneficial effect of VD on peripheral artery disease, foot ulceration, and wound healing. On the other hand, there is controversy about the effect of VD supplementation on cardiovascular adverse events, endothelial function, and eGFR improvement. Finally, the association of VDD with fragility fractures and depression in the elderly with T2DM remains controversial.

Discussion

Emerging evidence supports that VD is involved in glucose homeostasis and contributes to the pathophysiology of insulin resistance and diabetes mellitus. The exact pathophysiology is not clear yet, but the existing evidence suggests that VD improves beta-cell function (improvement of molecular repair mechanisms) and insulin sensitivity (reduction of oxidative damages, suppression of inflammatory response, and promotion of insulin signal transduction) [39].

All the epidemiological studies [5-11] (total seven studies; six retrospective and one meta-analysis) retrieved for this review correlate clearly VDD with T2DM in this age group (>60 years old). All these studies observed a high rate of VDD in patients with T2DM, while they also revealed inverse correlations between vitamin D status and metabolic control (HbA1c) and insulin resistance. It is worth noting that one of them supports that VD sufficiency protects against T2DM development.

The results from the studies in which different therapeutic regimens of vitamin D supplementation were administrated to examine the effects on metabolic control and insulin resistance were conflicting. Five studies (three randomized control studies, one meta-analysis, and one prospective study) [12-15,22] revealed that VD supplementation improved endothelial function, increased insulin sensitivity and b-cell function, reduced oxidative stress, and improved metabolic parameters to elderly with T2DM. On the other hand, two randomized control studies [16-17] with a small number of participants (in total 77 subjects) concluded that VD supplementation in this patient group didn’t improve the metabolic profile (HbA1c or insulin resistance).

Among the remarkable findings is the role of VDD in the complications of T2DM in the elderly population. A large randomized control trial [18] (with 9,795 participants) confirmed that VDD is associated with microvascular and macrovascular disease events in elderly T2DM patients. Myocardial infarction and stroke constitute the macrovascular complications, while retinopathy, nephropathy, neuropathy, and peripheral artery disease are the main microvascular complications. As far as cardiovascular events are concerned, we found six relative studies. One prospective study [19] and one cross-sectional study [20] support that VDD is associated with increased risk of myocardial dysfunction, cardiovascular morbidity, and mortality in T2DM elderly patients. Although, a retrospective study [21] indicates that VD doesn’t have a direct effect on coronary heart disease, but may have an indirect effect mediated by cardiovascular risk factors, such as diabetes duration, age, and sex. The three randomized control trials for this topic are also conflicting. The first in 2008 [22] supports that VD supplementation with one large dose improves endothelial function in T2DM patients, while the last in 2019 [23] concludes that VD supplementation with 4000 IU/day did not affect lipid profile and cardiovascular risk in patients with stable T2DM. In 2010 another RCT [17] revealed that a high dose of VD supplementation in T2DM patients improved blood pressure and b-type natriuretic peptide levels, but not the endothelial function. One observational study [30] showed that VDD is associated with microvascular complications and insulin usage in elderly with T2DM. The microvascular complications are the following: peripheral artery disease (PAD), nephropathy, and retinopathy. PAD is a devastating complication of diabetes mellitus with significant impact on the patient's functionality and health costs. We found three studies concerning VDD in elderly patients with PAD and T2DM: one cross-sectional study [24], one retrospective [25], and one RCT [26]. All of them support that VDD is associated with increased risk of PAD and diabetic foot ulcer formation, while the RCT concludes that VD supplementation for 12 weeks can improve wound healing. Regarding diabetic nephropathy, two studies (one cross-sectional and one retrospective study) [27-28] showed that VDD is associated with decreased glomerular filtration rate (GFR) and in addition VD status can be used to identify patients at increased risk of developing nephropathy complications. Although, an RCT [29] supports that VD supplementation resulted in no significant improvement of eGFR at five years. We retrieved only one study [31] for retinopathy for this patient group, which showed that VDD is clearly related with severe diabetic retinopathy. Diabetic peripheral neuropathy (DPN) can be a quite painful and disabling complication in elderly with T2DM. The two studies (one meta-analysis and one cross-sectional) [32-33] for DPN revealed that VDD is strongly correlated with DPN, while the VD status might be used as a predictor of DPN in elderly with T2DM. Cognition dysfunction is a relatively new complication of diabetes mellitus with partially undefined pathophysiology. We found only one retrospective study [34] published in 2019, which indicates that VDD may predict poor cognitive performance in patients with T2DM. VDD has been recently correlated with depression, although a cross-sectional study [35] published in 2017 concerning elderly with T2DM concluded that VDD appeared not to be a contributing factor to higher depression scores in elderly with T2DM. Although, a cross-sectional study conducted in 2015 [36] showed that VD sufficiency is associated with higher self-reported diabetes treatment satisfaction and better diabetes-specific quality of life in elderly with T2DM. Fragility fractures (FFs) have a tremendous impact on elderly functionality, mental health, and physical activity. We identified four studies concerning FFs in elderly with T2DM and VDD. One cross-sectional study [37] revealed that VD levels below 20 ng/ml were associated with increased vertebral fracture risk in elderly men with T2DM. An observational study [30] showed that VDD and insulin treatment is associated with increased risk of FFs, while a retrospective study [5] concluded that elderly with T2DM and VDD accompanied with increased parathyroid hormone are associated with increased risk for FFs. On the other hand, a retrospective study published in 2015 [38] supports that there is no correlation between VDD and HbA1c or FFs in elderly with T2DM. 

## Conclusions

In the last 20 years there has been a tremendous increase in publications concerning vitamin D deficiency and diabetes mellitus type 2, although elderly patients (>60 years old) didn’t receive the required proportion of these studies. After the discussion of the results, we can easily understand that many issues of this topic are still unexplored.

Undoubtably, there is a definite correlation of VDD with T2DM in elderly patients. However, the effect of vitamin D supplementation on metabolic and lipid profile, oxidative stress, and the complications of T2DM in older patients require larger randomized controlled trials to confirm the thesis that vitamin D supplementation could be a possible intervention to improve insulin secretion and retard the progression of diabetes in type 2 diabetic subjects, reducing the upcoming complications. The definition of the exact threshold of vitamin D levels and the regimen of vitamin D supplementation is paramount of importance, although very difficult to be determined. Based on the small number of studies (excluding epidemiological studies and RCTs) of this patient group and the conflicting results in specific points of this topic, we strongly believe that there is an emerging need for new well-designed studies for the elderly population with T2DM and VDD.
